# Recent Advances in Understanding and Managing Tourette Syndrome

**DOI:** 10.12688/f1000research.7424.1

**Published:** 2016-02-09

**Authors:** Mary Ann Thenganatt, Joseph Jankovic

**Affiliations:** 1 Parkinson’s Disease Center and Movement Disorders Clinic, Department of Neurology, Baylor College of Medicine, Houston, Texas, USA

**Keywords:** Tourette syndrome, neurology, behaviour

## Abstract

Tourette syndrome (TS) is a neurologic and behavioral disorder consisting of motor and phonic tics with onset in childhood or adolescence. The severity of tics can range from barely perceptible to severely impairing due to social embarrassment, discomfort, self-injury, and interference with daily functioning and school or work performance. In addition to tics, most patients with TS have a variety of behavioral comorbidities, including attention deficit hyperactivity disorder and obsessive-compulsive disorder. Studies evaluating the pathophysiology of tics have pointed towards dysfunction of the cortico-striato-thalamo-cortical circuit, but the mechanism of this hyperkinetic movement disorder is not well understood. Treatment of TS is multidisciplinary, typically involving behavioral therapy, oral medications, and botulinum toxin injections. Deep brain stimulation may be considered for “malignant” TS that is refractory to conventional therapy. In this review, we will highlight recent developments in the understanding and management strategies of TS.

## Introduction

Tourette syndrome (TS) is a neurobehavioral disorder characterized by motor and phonic tics that begin in childhood or adolescence. Adult-onset tics usually represent recurrences of childhood tics, although there are some tic disorders, other than TS, that can initially manifest in adulthood ^1^. A tic is an abrupt and brief movement or sound that is often preceded by a local or generalized urge or some other premonitory sensation. Thus, tic is an example of a group of hyperkinetic movement and other motor disorders that are increasingly recognized to be associated with a sensory phenomenon ^2^. Tics can be classified as clonic (jerk-like), dystonic (sustained), tonic (isometric), and blocking (cessation of movement and speech). In addition to its brief, intermittent, and repetitive nature, other features of tics include suggestibility, suppressibility, and distractibility, which may lead to a wrong diagnosis of a tic as a “psychogenic” movement disorder ^3– 5^. There is no diagnostic test for TS, but the Diagnostic and Statistical Manual of Mental Disorders Fifth Edition’s definition of TS requires the presence of multiple motor and phonic tics with onset prior to age 18 and lasting at least 1 year ^6^.

The epidemiologic findings in TS vary depending on the population studied, methodology, and many other factors. Among 21 population-based prevalence studies, the pooled TS population prevalence was estimated to be 0.52% (95% confidence interval [CI]: 0.32–0.85) ^7^. In one study, based on cross-sectional structured diagnostic interviews in 1374 TS patients and 1142 TS-unaffected family members, the lifetime prevalence of comorbid disorders was 85.7%; 57.7% of the population had two or more psychiatric disorders ^8^. Furthermore, 72.1% of the individuals met the criteria for obsessive-compulsive disorder (OCD) or attention deficit/hyperactivity disorder (ADHD), and of other disorders, including mood, anxiety, and disruptive behavior, each was present in approximately 30% of the patients. The age at greatest risk for the onset of most comorbid psychiatric disorders was between 4 and 10 years of age. The median age of onset of TS was 6 years of age.

In this review, we discuss the most recent developments in our understanding of the underlying pathophysiology and pathogenesis of TS as well as management and novel therapeutic strategies.

## Pathophysiology

Various physiologic, imaging, and genetic techniques have been used to provide insights into the pathophysiology of TS. TS has been considered a developmental disorder, but evidence for this hypothesis has been lacking ^9, 10^. Using 3-Tesla structural neuroimaging, one study examined cortical thickness in 52 TS patients and matched controls and found reduced depth and thickness of the gray matter in the pre- and post-central and superior, inferior, and internal frontal sulci, which correlated with tic severity ^11^. Abnormal brain plasticity involving the motor cortex, basal ganglia, and brainstem has been suggested as a mechanism for altered motor control processing in TS ^12, 13^.

Functional imaging and animal studies have pointed to TS as a network disorder with dysfunction in the cortico-striato-thalamo-cortical circuit ^9, 14, 15^. For example, one functional magnetic resonance imaging (fMRI) study evaluated 36 adult TS patients and found that temporal progression of structures involved during the generation of a tic followed a sequence of activation in the cortico-striato-thalamo-cortical circuit ^16^. Thus, 1–2 seconds before a tic, the supplementary motor area (SMA), ventral primary motor cortex, primary sensorimotor cortex, and parietal operculum exhibited activation, followed by the anterior cingulate, putamen, insula, amygdala, cerebellum, and the extrastriatal-visual cortex. During the onset of the tic, the thalamus, central operculum, and primary motor and somatosensory cortices became activated. Multimodal imaging techniques in 16 TS patients found that functional connectivity between anterior insular and sensorimotor cortex was increased ^17^. Diffusion tensor imaging techniques have shown reduced connectivity of frontal brain networks involved in planning and controlling actions in TS patients ^18^.

The cause of the disruption of the cortico-basal ganglia structures is not fully understood. Abnormalities of the γ-aminobutyric acid (GABA) system have been proposed as contributing to the disinhibition seen in TS. The use of positron emission tomography and [ ^11^C]flumazenil, a GABA receptor ligand, has identified abnormalities in the GABAergic system in TS patients, including decreased GABA-A receptor binding in the ventral striatum, globus pallidus, thalamus, amygdala, and right insula and increased binding in the bilateral substantia nigra, left periaqueductal grey, right posterior cingulate cortex, and bilateral cerebellum ^19^.

A major unmet need in TS research is clinical-pathological correlation. One neuropathological study investigated the density of interneurons and medium spiny neurons in the striatum of five TS brains compared to normal controls ^20^. In TS brains, there was a 50–60% reduction in cholinergic and parvalbumin interneurons, the latter representing inhibitory GABAergic interneurons in the striatum.

Studies of the urge, or premonitory sensation, associated with tics have identified neural networks associated with these sensory phenomena ^2^. Abnormalities in the sensorimotor cortex and somatosensory cortex have been found to be associated with the premonitory sensation ^9^. Imaging studies have suggested that areas outside of the sensorimotor network, such as the insula, also contribute to this urge. In one study using resting-state fMRI in 13 TS patients and 13 controls, the investigators found increased connectivity between the right dorsal anterior insula and the frontal-striatal nodes of the urge-tic network and the bilateral SMA which correlated with urge severity, thus suggesting that the anterior insula is involved in interoceptive awareness of sensations leading to the urge to tic ^21^.

## Genetics

While TS is clearly a familiar disorder, often with bilineal inheritance (both parents affected) ^22^, our understanding of the genetic underpinnings of TS is still in its infancy ^23^. A population-based study in Sweden found overall heritability of tic disorders to be 0.77, increasing with the degree of genetic relatedness: odds ratio (OR) of first-degree relatives (18.69) greater than that of second degree relatives (OR 4.58), which was greater than that of third-degree relatives (OR 3.07) ^24^. Despite the strong heritability of TS, a causative gene or genes have yet to be discovered ^25^. Various study designs have evaluated the genetics of TS including twin studies, linkage analyses, cytogenic abnormalities, copy number variation studies, and genome-wide association studies (GWAS) ^23^. Studies of multigenerational pedigrees of TS families have found an association between a functional mutation in the histamine decarboxylase ( *HDC*) gene and TS ^26, 27^. The *HDC* gene encodes for L-histidine decarboxylase, which is the rate-limiting step enzyme in histamine production. The first GWAS in TS included 1285 cases and 4964 controls of European ancestry ^28^. In the primary analysis, no markers achieved a genome-wide threshold of significance (p<5×10 ^-8^); the strongest signal was found in rs7868992 on chromosome 9q32 within COL27A1 (p=1.85×10 ^-6^). In a subsequent study, 42 single nucleotide polymorphisms (SNPs) (p<10 ^-3^) from this GWAS were genotyped in 609 additional cases and 610 controls ^29^. One SNP, rs2060546, was significantly associated with TS in this sample (p=3.3×10 ^-4^, OR=2.41) and when analyzed with the original GWAS data, this association was even greater (p=5.8×10 ^-7^, OR=1.77). This SNP, rs2060546, is located on chromosome 12q22 close to *NTN4*, a gene that codes for a protein in the developing striatum involved in axon guidance and outgrowth. In an attempt to examine the contribution of large, rare copy number variants to TS and OCD susceptibility, a genome-wide, cross-disorder study in 2699 cases (1086 TS, 1613 OCD) and 1789 controls found deletions in 16p13.11 ^30^. This suggests that mutations in this region may be associated with increased susceptibility to TS, OCD, and possibly other neurodevelopmental disorders. Based on a GWAS in 2723 cases (1310 with OCD, 834 with TS, and 579 with OCD plus TS), 5667 ancestry-matched controls, and 290 OCD parent-child trios, no individual SNPs achieved genome-wide significance ^31^. However, polygenic score analyses identified a significant polygenic component for OCD but not for TS. The study concluded that “OCD with co-occurring TS may have different underlying genetic susceptibility compared with OCD alone”.

## Pharmacologic therapy

Dopamine receptor blocking drugs (neuroleptics) and dopamine-depleting drugs have been traditionally used in the treatment of TS even though functional imaging studies or postmortem studies regarding striatal dopaminergic hyperinnervation in TS have been inconclusive ^32^. Although the only two approved therapies for TS by the U.S. Food and Drug Administration (FDA) are haloperidol and pimozide, many other agents are currently used or are in development for the treatment of TS ^33, 34^. While the benefit of haloperidol and pimozide for the treatment of TS has been demonstrated in multiple trials, their use is limited by their short- and long-term side effects, including sedation, weight gain and other metabolic complications, hyperprolactinemia, acute dystonic reactions, parkinsonism, tardive dyskinesia, and neuroleptic malignant syndrome ^35^. Other neuroleptics with potentially fewer side effects have been studied in TS. Fluphenazine, a typical antipsychotic with D1- and D2-receptor blocking activity, has also been shown to be effective for TS in small studies. A recent retrospective review of the long-term efficacy and safety of fluphenazine for the treatment of TS was conducted at Baylor College of Medicine from 1985 to 2011 ^36^. This study included 268 TS patients treated with fluphenazine for an average of 2.6 ± 3.2 years (range 0.01–16.8, 40 patients over 5 years and 13 patients over 10 years). The mean age of initiation was 15.8 ± 10.8 years (range 4.1–70.2) and the mean dose at last follow-up was 3.2 ± 2.3 mg (range 0.5–12 mg) per day. Response to fluphenazine was rated as “moderate to marked” in 80.5% of patients at initial visit and 76% at last follow-up. The most common side effects were drowsiness (26.1%), weight gain (11.6%), akathisia (8.5%), and acute dystonic reactions (7.0%); there were no cases of tardive dyskinesia.

Atypical antipsychotics such as risperidone and aripiprazole have been studied in open-label and randomized, placebo-controlled studies for the treatment of TS ^35^. A meta-analysis of antipsychotics for the treatment of TS found no difference in efficacy among risperidone, haloperidol, pimozide, and ziprasidone ^37^. Side effects such as sedation, weight gain, and hyperprolactinemia have limited their tolerability ^35^. Aripiprazole, a third-generation antipsychotic with D2-receptor antagonist and agonist properties, has been studied for TS in case series and open label studies. The largest case series of aripiprazole for the treatment of TS included 100 patients treated between 2005 and 2010 ^38^. These patients (mean age of 27.1 ± 11.5 years) were treated with a mean daily dose of 17.0 ± 9.6 mg. At first follow-up after 1–6 months, 82% had moderate or marked reduction in tic severity and this was sustained in all but one patient after 12-month follow-up. Five patients reported an improvement in comorbid psychiatric disorders including anxiety, depression, aggression, and ADHD, which may be partly due to aripiprazole’s effect on the serotonergic system. Adverse effects were reported in 51% of subjects, with the most common being drowsiness, agitation, sleep disturbance, and nausea. A randomized, double-blind, placebo-controlled trial of aripiprazole included 61 children and adolescents with TS ^39^. After 10 weeks, there was significantly greater improvement in the Yale Global Tic Severity Scale (YGTSS) total tic score compared to baseline in the treatment arm compared to placebo (-15.0 vs. 9.6, respectively, p=0.0196). Side effects of aripiprazole included significant increase in mean body weight, body mass index, and waist circumference. A systematic review of aripiprazole for TS included six randomized controlled trials and found a similar efficacy of aripiprazole and haloperidol for TS but with significantly less risk of drug-induced movement disorders with aripiprazole (1.5%) vs. haloperidol (43.5%) ^40^. The most common adverse effects attributed to aripiprazole were nausea/vomiting (5.5%), drowsiness (3.9%), headache (3.5%), and dizziness (3.5%). Although the drug has been promoted as having a low risk of tardive dyskinesia, this iatrogenic complication has been reported with all typical and atypical neuroleptics (with a possible exception of clozapine), including aripiprazole ^41^.

In addition to neuroleptics approved by the FDA for the treatment of psychiatric disorders, there are novel, experimental, anti-dopaminergic drugs currently being investigated in the treatment of TS. Ecopipam, a selective D1-receptor antagonist, was evaluated in 18 adult TS patients in an open-label study ^42^. There was significant improvement in the primary endpoint, a change in YGTSS total tic score at 8 weeks (25.3) compared to baseline (30.6) (p=0.0004). The most common adverse events were sedation (39%), fatigue (33%), insomnia, (33%), somnolence (28%), anxiety (22%), headache (22%), and muscle twitching (22%). This drug has the potential to improve tics, but it is not yet clear whether its side effects will include those typically associated with D2-receptor blockade, including tardive dyskinesia. A multi-center, randomized, placebo-controlled study of ecopipam in children with TS is currently underway in several North American centers (ClinicalTrials.gov registration: NCT02102698).

Tetrabenazine, a dopamine-depleting drug that acts by inhibiting vesicular monoamine transporter 2 (VMAT2), has been shown to be effective in open-label trials for the treatment of TS ^43, 44^. A retrospective study conducted at Baylor College of Medicine evaluated the response to tetrabenazine in patients with various hyperkinetic movement disorders including 92 with tics (mean age 24.1 years) ^43^. Moderate to marked improvement in tics was noted by 76.7% of patients. The most common adverse effects reported, all dose-related, were drowsiness (25.0%), parkinsonism (15.4%), depression (7.6%), and akathisia (7.6%). An open-label prospective study of tetrabenazine for TS included 120 patients ^45^. Patients received a mean daily dose of 70.5 mg for a mean duration of 19 months. There was improvement in the Clinical Global Impression of Change scale in 76% of patients at the final visit and side effects occurred in 2–5% of patients (somnolence, depression, asthenia, parkinsonism, and akathisia). In the United States, tetrabenazine (now available as a generic drug) carries a black-box warning regarding the risk of depression and suicidality. This potential side effect seems to be more common in patients with a pre-existing history of depression ^46^. In contrast to the dopamine receptor blocking drugs, tetrabenazine does not appear to cause tardive dyskinesia and may have fewer other side effects, including weight gain ^47^. Other dopamine depletors currently being investigated in the treatment of TS include SD-809 (deutetrabenazine), a deuterated form of tetrabenazine, which is also being investigated in tardive dyskinesia and Huntington disease (Clinicaltrials.gov registration: NCT01795859), and NBI-98854, purified parent drug of the (+)-α-isomer of tetrabenazine (valbenazine), (Clinicaltrials.gov: NCT02581865). In a pilot, open-label study, 23 adolescent patients (mean age 16 years; range: 12–18) with moderate-to-severe tics associated with TS were titrated over a 6-week period and maintained for 2 weeks at a mean dose of 32.1 mg (range: 18–36 mg) of SD-809 ^34^. An independent blinded rater assessed tic severity using the YGTSS and tic impact using the TS-Clinical Global Impression. The mean YGTSS total tic score improved by 37.6% (p<0.0001) and there were also significant improvements in secondary outcome measures. No serious or severe adverse effects were reported, but one subject withdrew from the study for an adverse effect of irritability that was unrelated to the study drug. These VMAT inhibitors, SD-809 and NBI-98854, have a more favorable pharmacokinetic and side effect profile than tetrabenazine, as suggested by some preliminary studies in Huntington disease and tardive dyskinesia, although further studies are needed.

A variety of antiepileptic medications have been studied in TS ^48^. A meta-analysis of topiramate for TS identified 14 trials involving 1003 TS patients ^49^. The included studies had either haloperidol (12 studies) or tiapride (two studies) as the control. There were many limitations to this analysis, including overall poor quality of the randomized trials. A meta-analysis of three trials that used the YGTSS to evaluate tic severity found a significant improvement in this scale favoring topiramate compared to control treatment. In a meta-analysis of nine studies evaluating improvement of tics by >50%, there was no significant difference between the topiramate and control groups. A multi-center, placebo-controlled study of topiramate in TS involving 29 patients (mean age of 16.5 years) showed a significantly greater improvement in the total tic score of the YGTSS at day 70 compared to baseline in the topiramate group (mean dose 118 mg) vs. placebo (-14.29 vs. 5.00, p=0.0259) ^50^. There was also significant improvement in the clinical global impression and premonitory urge without a difference in adverse events between the groups. Levetiracetam, another anticonvulsant, has been shown to be helpful in the treatment of tics in open-label studies, but randomized controlled studies failed to demonstrate its benefit ^48^.

Alpha agonists such as guanfacine and clonidine have been found to be useful in the treatment of mild tics and may have a particular benefit in patients with co-existing ADHD and impulse control disorder ^37^. A meta-analysis of published studies of alpha-2 agonists for the treatment of TS found a medium-to-large effect on tics in subjects who had co-existing ADHD, but only a small non-significant benefit on tics in studies that excluded co-existing ADHD ^37^. Potential side effects of these medications include sedation, light-headedness, headaches, and irritability.

Botulinum toxin may be helpful in the treatment of focal motor tics and in some simple and complex phonic tics (including coprolalia) ^51^. Open-label studies and cases series have demonstrated the benefits of botulinum toxin not only in ameliorating the intensity of the tics but also in reducing the frequency and the regional premonitory urge ^52^. A randomized, double-blind, placebo-controlled study of botulinum toxin for motor tics demonstrated a significant improvement in urge and tic frequency with botulinum toxin ^53^. This small study showed no significant differences in other measures, such as severity score, tic suppression, pain, and patient global impression, possibly because of its small sample size, relatively mild symptoms, and a single treatment protocol which does not reflect the clinical practice of evaluating patients after several adjustments in doses and sites of injections. A report of 30 TS patients treated with botulinum toxin for phonic tics reported an improvement in 93% of patients with 50% being free of phonic tics ^54^. Premonitory sensation and quality of life also improved. The most common side effect was hypophonia in 80% of patients. Botulinum toxin can be helpful in targeting a few particularly bothersome tics that are refractory to oral medications, such as repetitive cervical extension (so called “whiplash tics”) seen in some cases with malignant TS that can be associated with subsequent complications such as compressive cervical myelopathy ^55^.

There is a growing public interest in cannabinoids for the treatment of movement disorders including TS ^56^. Anecdotally, patients often report an improvement in tics and some behavioral symptoms with cannabis ^57, 58^. In two small controlled trials, an improvement in tics was demonstrated with delta-9-tetrahydrocannabinol (THC) without major side effects ^57^. According to a Cochrane review on the efficacy of cannabinoids in TS, definitive conclusions about the safety and efficacy of cannabinoids in the treatment of TS cannot be drawn ^59^. Further studies with a larger number of patients and of longer duration are needed to determine the efficacy and safety of cannabinoids for the treatment of TS ^56^.

New therapies for TS are being studied in clinical trials including AZD5213, a histamine H3 receptor antagonist. Given the possible association between mutations in the *HDC* gene and TS, this drug may be promising for TS.

## Behavioral therapy

Behavioral therapy is another treatment option for TS and its efficacy has been demonstrated in a number of randomized controlled trials. One study compared behavioral therapy to supportive therapy and education (control group) in children aged 9–17 years with TS or chronic tic disorder ^60^. This study included 126 children who were randomized to eight treatments of behavioral therapy over 10 weeks or control group. Three monthly booster sessions were conducted for those who responded. The behavioral therapy consisted of comprehensive behavioral intervention for tics (CBIT), which is based on habit reversal therapy and includes other components such as relaxation techniques. A blinded examiner evaluated the subjects. After 10 weeks of treatment, the behavioral intervention resulted in a significantly greater reduction in the YGTSS (24.7 to 17.1) from baseline to endpoint compared with the control treatment (24.6 to 21.1, p<0.001) with a difference between groups of 4.1. On the Clinical Global Impression–Improvement Scale, the behavioral intervention group was more likely to be rated as “very much improved” or “much improved” compared to the control group (52.5% vs. 18.5%, p<0.001). Only 12/126 subjects did not complete the study. These outcomes were independent of tic severity. Of the initial responders who were evaluated at 6 months, 87% had a persistent response to behavioral therapy. A second randomized controlled trial evaluated the benefit of CBIT, this time in adult patients with TS or chronic tic disorder ^61^. In this study, 122 subjects were randomized to eight sessions of CBIT or eight sessions of supportive therapy over 10 weeks. At 3 months, there was significantly greater mean reduction compared to baseline in the YGTSS in the CBIT group (24.0 to 17.8) compared with the control group (21.8 to 19.3) (p<0.001). In the treatment group, 38.1% was rated as “much improved” or “very much improved” on the Clinical Global Impression–Improvement scale vs. 6.4% in the control group. The dropout rate was 13.9%, and for those available for assessment, there was persistent benefit at 6 months. A 2014 meta-analysis of the randomized controlled trials of behavioral therapy in TS identified eight trials with a total of 438 participants ^62^. There was a medium-to-large effect size for behavioral therapy compared to comparison conditions, and the number needed to treat was three. Increasing age, a greater number of treatment sessions, and less co-occurring ADHD was associated with a greater effect size. While this therapeutic intervention may be helpful for certain patients, as with all treatment options for TS, there are some limitations to this form of therapy, including the amount of effort and compliance by the patient (and parents) needed for this therapy to provide sustained benefit, limited access to providers trained in the technique, and lack of insurance coverage for the treatment ^63^.

## Surgical therapy

Deep brain stimulation (DBS) can be effective for patients with medication and behaviorally refractory TS with disabling tics. In one retrospective study, which included 13 patients with refractory TS treated with globus pallidus internus (GPi) DBS at follow-up of a mean 41.9 months (range 13–80 months), there was a 52.1% improvement in YGTSS compared to baseline ^64^. Furthermore, there was mean improvement of 45.7% in the Gilles de la Tourette Syndrome-Quality of Life Scale score. In another study involving 48 patients with refractory TS treated with mostly thalamic DBS, in which 37 subjects completed the study, 78% had a reduction of greater than 50% on the YGTSS ^65^. A double-blind, crossover trial enrolled 15 patients with refractory TS to evaluate GPi DBS ^66^. Subjects were randomized to stimulation on or off for the first 3 months and then switched to alternative treatment. The patients and evaluating clinicians were blinded to the treatment group. There was a mean improvement of 12.4 points (p=0.048) on the YGTSS total score in the on-stimulation period compared to off-stimulation period. During the open-label phase, there was a greater improvement in YGTSS total scores, possibly reflecting the influence of patient expectations on outcomes. There were three serious adverse events, two hardware infections, and one DBS-induced hypomania.

The two most common targets studied for DBS in TS are the thalamus (centromedian parafascicular complex, centromedian nucleus-substantia periventricularis-nucleus ventro-oralis internus) and the postero-ventrolateral and antero-medial regions of the GPi ^67^. However, multiple other targets have been tried including the anterior limb of the internal capsule, nucleus accumbens, subthalamic nucleus, and the globus pallidus externus. Potential side effects of DBS in general include intracerebral hemorrhage, ischemia, and infection. There have been reports of higher rates of infection (18%) in DBS for TS thought to be due to compulsive touching of the surgical scar in TS patients ^68^. Psychiatric symptoms are also possible, including worsening depression, hypomania, psychosis, anxiety, and agitation ^67^. A small study involving five TS patients demonstrated the feasibility and improvement of “scheduled” DBS at regular intervals for tic severity ^69^. This was a proof-of-concept study of scheduled DBS therapy as opposed to the classic continuous stimulation. Future techniques of neuromodulation may focus on closed-loop stimulation that adjusts stimulation parameters based on neural feedback and thus providing more effective and efficient therapy ^70^. A panel of experts reviewed 48 studies of DBS for TS published since 1999 and proposed consensus guidelines ^71^. The recommendations of the panel were as follows: 1. DBS should be considered in TS patients with disabling tics with a YGTSS >35/50; 2. patients should have tried medications from at least three pharmacologic categories − alpha-adrenergic agonists, dopamine antagonists, and one additional category; and 3. behavioral therapy, such as CBIT, should be offered to patients prior to surgery. Patients should be evaluated by a multidisciplinary team, and comorbid conditions should be treated and stable.

The ideal DBS target in TS is still unknown ^72^. The findings of randomized controlled studies of DBS in TS are difficult to assess because of the fluctuating nature of tics, the lack of standardized programming strategies, and the relatively long duration needed for tic control ^68, 73^.

The optimal treatment strategy for TS patients must take into consideration the severity of tics and their effect on daily functioning and quality of life ( Table 1 and Table 2) ^34, 74^. Behavioral therapy may be limited by access to trained providers. Various pharmacologic treatments including oral medications and botulinum toxin should be selected based on tic severity and comorbid conditions, keeping side effect profiles of these medications in mind. Finally, DBS should be reserved for those with severe tics that are refractory to conventional therapy.

**Table 1.** Treatment options for Tourette Syndrome.

*Alpha agonists* Clonidine Guanfacine *Antiepileptics* Topiramate *Dopamine Depletors* Tetrabenazine *Antipsychotics* Fluphenazine Aripiprazole Risperidone Pimozide Haloperidol *Botulinum Toxin* *Behavioral Therapy* Comprehensive Behavioral Intervention for Tics (CBIT) *Deep Brain Stimulation* Thalamus Globus Pallidus Interna

**Table 2.** Emerging Therapies for Tourette Syndrome.

*Ecopipam* D1 receptor antagonist *SD-809* Deutetrabenazine (VMAT2 inhibitor) *NBI-98854* Valbenazine (VMAT2 inhibitor) *AZD5213* Histamine H3 receptor antagonist *Cannabinoids* *New DBS targets* Nucleus accumbens, subthalamic nucleus, and the globus pallidus externus

VMAT2 = vesicular monoamine transporter 2

DBS = deep brain stimulation

## Conclusions

TS is a complex disorder with motor manifestations (motor and phonic tics) and a variety of behavioral co-morbidities (ADHD, OCD, impulse control disorders, and others). While there have been advances in our understanding of the neural networks involved in TS, particularly as a result of new imaging techniques, the mechanisms of the disturbed pathways are still not well understood. Furthermore, despite a strong heritability for the disorder, a causative gene or genes have yet to be identified. Various treatment options are available for TS and need to take into consideration tic severity, effect on quality of life, and comorbid disorders. Further study of DBS for medically refractory malignant TS is needed in order to determine the most effective target and optimal programming strategies. New medical and surgical treatments are currently being studied in order to provide better quality of life in patients with TS.

## Abbreviations

ADHD = attention deficit/hyperactivity disorder

CI = confidence interval

CBIT = comprehensive behavioral intervention for tics

DBS = deep brain stimulation

FDA = Food and Drug Administration

fMRI = functional magnetic resonance imaging

GABA = γ-aminobutyric acid

GPi = globus pallidus internus

GWAS = genome-wide association study

HDC = histamine decarboxylase

OCD = obsessive-compulsive disorder

OR = odds ratio

TS = Tourette syndrome

SMA = supplementary motor area

SNPs = single nucleotide polymorphisms

VMAT2 = vesicular monoamine transporter 2

YGTSS = Yale Global Tic Severity Scale
